# Genetic diversity and matrilineal genetic origin of fat-rumped sheep in Ethiopia

**DOI:** 10.1007/s11250-019-01827-z

**Published:** 2019-02-24

**Authors:** Helen Nigussie, Joram M. Mwacharo, Sarah Osama, Morris Agaba, Yoseph Mekasha, Kefelegn Kebede, Solomon Abegaz, Sanjoy Kumar Pal

**Affiliations:** 1 Present Address: Department of Microbial, Cellular and Molecular Biology, Addis Ababa University, P.O. Box 1176, Addis Ababa, Ethiopia; 2 International Center for Agricultural Research in the Dry Areas (ICARDA) c/o ILRI, P.O. Box 5689, Addis Ababa, Ethiopia; 3 Biosciences eastern and central Africa (BecA-ILRI), International Livestock Research Institute, P.O. Box 30709 00100, Nairobi, Kenya; 4 The Nelson Mandela African Institution of Science and Technology (NM-AIST), P.O. Box 447, Arusha, Tanzania; 5 The Agricultural Transformation Agency, Addis Ababa, Ethiopia; 6 Institute of Agricultural Sciences in the Tropics, Department of Animal Breeding and Husbandry in the tropics and Sub tropics- 490h, University of Hohenheim Garrbenstr, 17/70599 Stuttgart, Germany; 7 Ethiopian Institutes of Agricultural Research, P.O. Box 32, Debre Zeit, Ethiopia; 8 School of Pharmaceutical & Allied Medical Sciences, School of Natural Sciences, CT University, Jagraon Ludhiana, Punjab 142024 India

**Keywords:** Microsatellites, mtDNA D-loop sequence, *Ovis aries*

## Abstract

Ethiopia is home to a diverse gene pool of indigenous sheep populations. Therefore, a better understanding of genetic variation holds the key to future utilization through conservation. Three of these breeds, Afar, Blackhead Somali, and Hararghe Highland, are found in eastern Ethiopia where they contribute significantly to the livelihood of most pastoralist, agro-pastoralist, and smallholder farmers. These indigenous sheep are recognized on the basis of morphotype and their genetic distinction remains unknown. Here, to assess genetic variation, and matrilineal genetic origin and relationship of fat-rumed sheep found in eastern Ethiopia, 300 individuals from the three breeds were genotyped for 22 microsatellite markers and sequenced for the mitochondrial DNA displacement loop (mtDNA d-loop) region. The overall *H*_O_ and *H*_E_ were 0.57 and 0.75, respectively. Differentiation statistics revealed that a high proportion (97%) of the total genetic variation was explained by differences between individuals within populations. Genotype assignment independent of the population of origin showed *K* = 2 to be the optimum number of genetic backgrounds present in the dataset. This result was further confirmed by mtDNA D-loop sequences comparison in which the matrilineal genetic origin of eastern Ethiopia sheep is from two haplotype groups (types A and B) among the five haplotypes globally observed. Taken together, our findings suggest that the sheep populations from three breeds originated from two ancestral genetic backgrounds that may have diverged prior to their introduction to Ethiopia. However, to obtain a complete picture of the evolutionary dynamics of Ethiopian indigenous sheep, more samples and populations from within and outside of the country will need to be analyzed.

**Electronic supplementary material:**

The online version of this article (10.1007/s11250-019-01827-z) contains supplementary material, which is available to authorized users.

## Introduction

Animal genetic resources are critical for global food security and livelihoods. Livestock products have high densities of energy, protein, and other critical nutrients, which are necessary for infants and expectant mothers (Boettcher et al. 2014). Furthermore, there is a huge demand for animal products in developing countries, where most demand is met by local production (Thornton 2010). In Ethiopia, changes in the demand for livestock products have been driven by human population growth, income increment, and expansion of urbanization. Along with this, a large export and domestic market for mutton and live animal has created opportunity for sheep production in Ethiopia. Besides, the strategic location of Ethiopia to the Middle East is also an opportunity to export meat (largely from sheep and goats) and live animals to these countries. There are about 27.3 million sheep in Ethiopia, out of which 99.9% are indigenous breeds (CSA 2017) which are owned and managed by smallholder farmers and pastoralists under traditional and extensive production systems. The major sources of sheep exports (live animal and mutton) are from eastern lowlands of Ethiopia. In eastern Ethiopia, the Afar (AFR) and Black Head Somali (BHS) sheep are the predominant fat-rumped sheep populations that are distributed in arid and semi-arid areas (Gizaw et al. 2007), while Hararghe highland (HHL) sheep is also found in the highland where a crop-livestock mixed farming system is practiced (Shibabaw et al. 2014). The same sheep populations have been known for their adaptive traits such as resistance to disease, utilization of low-quality feed, and survival in harsh environments (tolerance to drought) (Nigussie et al. 2015).

According to Gizaw et al. (2008), BHS and AFR breeds were shortlisted as threatened among the eight of sheep. This calls for pragmatic actions to characterize, conserve, and sustainably use these breeds. The loss of genetic variation within and between breeds is detrimental not only from the perspectives of the culture of conservation and investigation, but also for utility since lost genes may be of future economic interest (FAO 2000). Once animal genetic diversity has been lost it cannot be replaced (FAO 2000). Besides, live animal trade with unsustainable rates of off-take, recurrent drought, and introduction of crossbred sheep by Ethiopian sheep genetic improvement program (ESGIP), USAID, and government of Ethiopia in the area will contribute to the loss of genetic diversity unless an appropriate measure is taken.

A limited effort has been made to understand within- and between-population levels of genetic variation in AFR and BHS sheep using microsatellite markers (Gizaw et al. 2007), but no attempt has been done to identify genetic diversity and the origin of HHL sheep so far. The maternal genetic origins of these populations were also not known though they are distributed in other neighboring countries. However, understanding within-population variation, their relationship, and uniqueness with other populations using appropriate and reliable molecular characterization tools have a paramount importance in developing sustainable breeding and conservation programs. The objectives of this study were to characterize the genetic diversity and population structure of fat-rumped sheep populations in eastern Ethiopia and to assess matrilineal genetic origin and genetic relationships among eastern Ethiopian sheep populations. Information from detailed molecular characterization will be used as an input to design sustainable genetic improvement strategy to satisfy the growing export demand for mutton along with sustainable conservation program.

## Material and methods

### Description of the study site

**Fig. 1:**
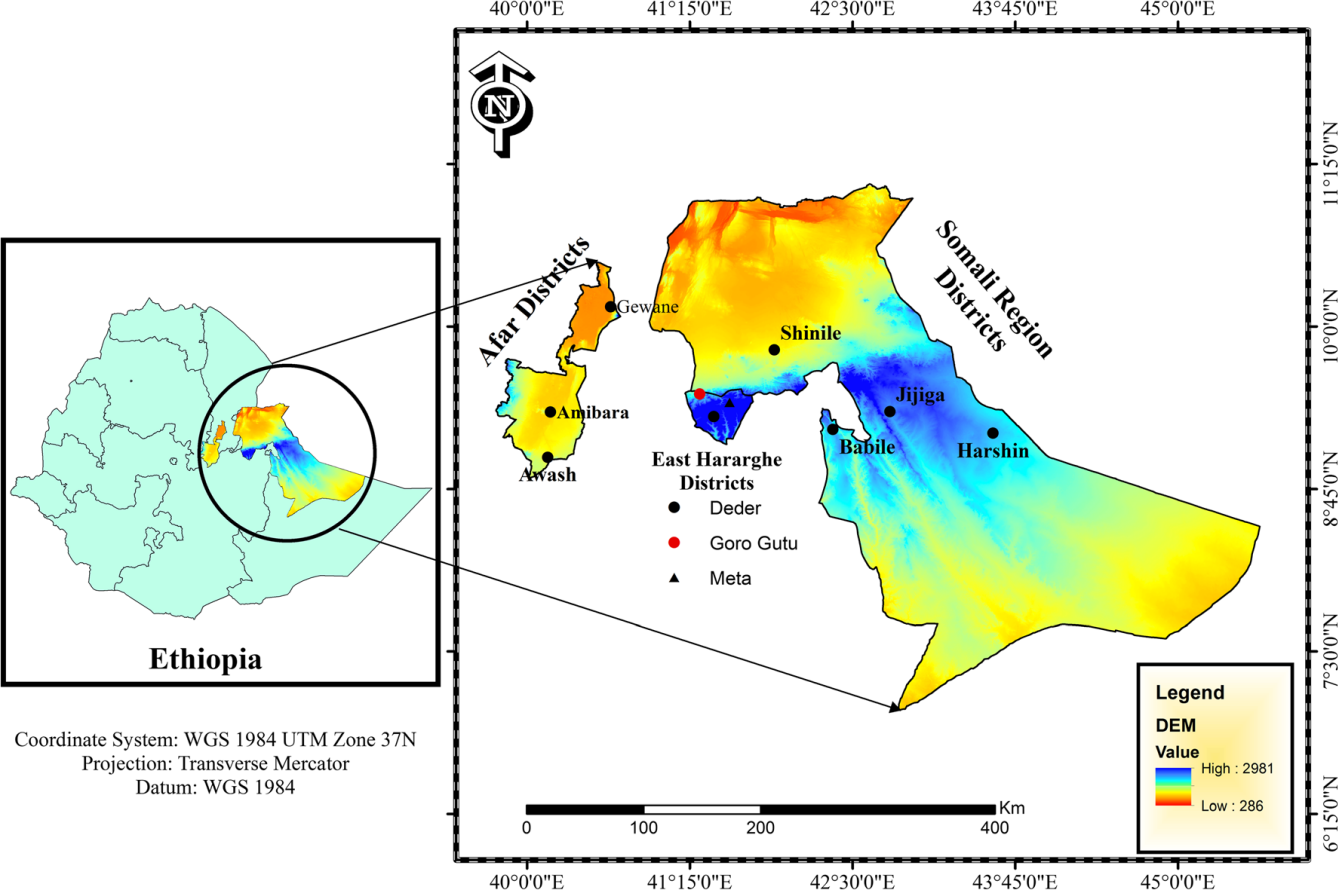
Map of the study area and sampling sites (•) for blood samples collection

The study was conducted in eastern Ethiopia, Jijiga and Shinile (Somali National Regional State), east Hararghe (Oromia National Regional State), and Gabi Rasu (Administrative Zone 3 of Afar National Regional State) (Fig. 1). Description of sampled sheep populations with respective production system is indicated in Table S1. Except for the Hararghe area which is a highland and with a moderate level of annual rainfall, the others are lowland areas with arid and semi-arid climatic conditions. Both AFR and BHS are managed by Afar and Somali pastoralist, respectively, while HHL sheep is managed by smallholder farmers who practice a mixed crop-livestock farming system.

### Blood sample collection

To capture the wide spectrum of the genetic diversity of the three populations, a total of ten locations were considered (Table S1). For each sampling site, 30 animals from different villages and flocks (two to three animals per flock) were sampled. A total of 300 blood samples were collected from the jugular vein using 5-ml ethylenediaminetetraacetic acid (EDTA) vacutainer tubes. Only adult ewes having two or more than two pairs of permanent incisors were considered in this study due to the shortage of matured ram in most of the study areas. The collected blood samples were transferred into Whatman FTA® Classic Card and transported to the International Livestock Research Institute Biosciences central and eastern Africa (ILRI-BecA hub), Nairobi, Kenya, for DNA extraction and further laboratory analysis.

### Microsatellite markers and genotyping

Twenty-two fluorescent-labeled microsatellite markers recommended by the ISAG/FAO to be used for sheep genetic diversity studies were genotyped (Table S2) (FAO, 2011). The forward primers of each pair were labeled with NED, FAM, and VIC dyes (Applied Biosystems, Warrington, UK). Genomic DNA was isolated from blood spotted on Whatman® FTA® cards (GE Health Care Life Sciences) following the manufacturer’s instructions. Polymerase chain reactions (PCR) were carried out in 10-μl reaction volumes containing 2 μl of DNA template (20–30 ng), 1 μl buffer, 0.2 μl dNTPs (2 μM), 0.8 μl of each primer (10 pmole), 0.08 μl of Taq (Platinum, Invitrogen), 1 μl of BSA (bovine serum albumin), and nuclease-free water to make the final volume. Touchdown PCR was carried out using the GenAmp® PCR System 9700 (Applied Biosystems) with the following condition: initial denaturation at 94 °C for 3 min followed by 35 cycles of 30 s at 94 °C, 30 s at annealing temperatures (ranged from 50 to 55 °C, 55 to 60 °C, and 60 to 65 °C depending on the primer (Table S2)), and 30 s at 72 °C. A final extension at 72 °C for 5 min completed the reactions. Amplified PCR products were multiplexed and analyzed by capillary electrophoresis using the automated ABI Prism® 3100 (Applied Biosystems, Foster City, CA, USA) and LIZ 350 internal lane size standard (Applied Biosystems). The data were collected and analyzed to determine the size of the microsatellite fragments using the GeneScan software (version 3.1). The resultant data were then imported into the GeneMapper software version 3.7 (Applied Biosystems) for allele sizing at each microsatellite locus.

### Mitochondrial DNA displacement loop amplification and sequencing

To amplify the mitochondrial DNA displacement loop (mtDNA D-loop) region, primers were designed from the published ovine reference sequence (AF010406; Hiendleder et al. 1998). The primer sequences were tRNA-proline (5′-AGTGCCTTGCTTTGGTTAAGC-3′) and tRNA-phenyl alanine (5′-CACCATCAA-CCCCAAAGCTGAAG-3′). The sequencing product was about 1180 bp that spanned between sites 15,437 and 16,616 bp of the reference sequence. In total, the D-loop region was only sequenced in 31 samples, 14, 8, 9 for AFR, BHS, and HHL populations, respectively, due to low DNA quality and PCR product concentration (< 10 ng). The PCR was carried out in 25 μl reaction volumes containing 4 μl of DNA template (30–50 ng), 12.69 μl of nuclease-free water, 2.5 μl buffer, 0.4 μl dNTPs (2 μM) and MgCl_2_ (1.5 μM), 2.5 μl of each primers (10 pmole), 0.09 μl of Taq (Platinum, Invitrogen), and 0.32 μl of BSA (bovine serum albumin). PCR was carried out using the GenAmp® PCR System 9700 (Applied Biosystems) with the following conditions: initial denaturation at 94 °C for 5 min, followed by 35 cycles of amplification at 94 °C for 30 s, annealing at 62 °C for 30 s, extension at 72 °C for 1 min, and a final extension at 72 °C for 5 min. PCR products were purified using Invitrogen Pure Link PCR purification kit (Thermo Fisher Scientific) and sequenced for both strands at Sequencing Genotyping and Oligonucleotide (SEGOLIP) unit at the same laboratory mentioned above.

### Data analysis

#### Microsatellite loci

The total number of alleles (TNA), mean number of alleles (MNA), allelic richness (AR), polymorphic information content (PIC), effective number of alleles (ENA), and expected (*H*_E_) and observed (*H*_O_) heterozygosis were estimated from allele frequencies with FSTAT 2.9.3.2 (Goudet 2001) and POPGENE 1.32 (Yeh et al. 1999). Total genetic variation of the populations (*F*_IT_) was partitioned into within (*F*_IS_) and among population (*F*_ST_) components following Weir and Cockerham (1984). For each locus-population combination for the global data set and each population, we used Fisher’s exact test GENEPOP 4.6 (Rousset 2017). Exact *P* values were estimated using the Markov chain algorithm with 10,000 dememorizations, 500 batches, and 5000 iterations per batch and corrected for multiple tests using the Bonferroni method (Rice 1989). We used the Bayesian clustering algorithm implemented in STRUCTURE 2.3.3 (Pritchard et al. 2010) to infer population structure and explore the assignment of individuals and populations to specific genetic clusters. For this analysis, we allowed the number of clusters (*K*) to vary between 1 ≤ *K* ≥ 15, using a burn-in of 100,000 followed by 200,000 Markov Chain Monte Carlo (MCMC) iterations. Ten simulations were carried out for each *K* assuming populations are admixed, and allele frequencies are correlated. To estimate the most optimal *K*, the number of clusters (*K*) was plotted against DeltaK calculated as mean(|L”(K)|)/sd(L(K)) and the optimal number of clusters identified by the largest change in log-likelihood (L(K)) values between the estimated number of clusters (Evanno et al. 2005). DISTRUCT (Rosenberg 2004) was used to generate a graphical display of the simulated results. The possibilities of non-random associations between genetic differentiation, measured as [FST/(1 − FST)] (Rousset 1997), and geographic distances in kilometers, were tested using the IBDWS 3.05 (http://ibdws.sdsu.edu). Geographic distances between populations were calculated using Google map as the distance between the main towns within each of the sampling locations.

#### Mitochondria DNA D-loop sequences

Two mitochondria DNA (mtDNA) D-loop sequence data sets were used in this study. The first 31 sequences generated were used to estimate maternal genetic diversity and elucidate relationships between the eastern Ethiopia sheep populations. The second data set was retrieved from the Gene Bank which represent the five (A, B, C, D, and E) globally defined domestic sheep mtDNA D-loop haplogroups (DQ851886-91, DQ852225- DQ852259, DQ852250-DQ852261, DQ852274-75, and DQ852278-79, respectively). Electropherograms were visualized, edited, and aligned using the ClustalW (Thompson et al. 1994) and the MEGA 6.0 software (Tamura et al. 2013). All sequences were trimmed to the shortest one. Fragments of 925 base pairs (from np 15,452 to np16, 263) eventually resulted from the standardization of the sequencing results. Cluster analysis using the neighbor-joining algorithm (Saitou and Nei 1987) was also performed in MEGA 6.0. The robustness of the resulting dendrogram was checked by running 1000 bootstrap replications. The extent of mtDNA D-loop polymorphism represented by haplotype diversity (Hd), nucleotide diversity (π), number of haplotypes (nh), and number of segregating sites (*S*) was calculated using DnaSP v.5.10.01 (Librado and Rozas 2009) following Tajima (1983) and Nei (1987) procedures. An analysis of variance (AMOVA) was computed to test significant differences in mitochondrial diversity between eastern Ethiopia populations using ARLEQUIN 3.01 (Excoffier, et al. 2005). Median-joining network (Bandelt et al. 1999) was drawn from haplotypes using the program Network 4.1.0.9 (www.fluxus-engineering.com). The maximum parsimony (MP) calculation was used to remove unnecessary median vectors and to avoid reticulations, which could be switched off in the results display.

## Results

### Within-population autosomal genetic diversity

A total of 255 alleles were detected at the 22 microsatellite loci assayed in three populations across 10 locations of eastern Ethiopia. The total number of alleles per locus ranged from 3 at BM1824 to 23 at SRCRSP8 (Table 1). The average PIC value across the 22 loci was 0.69 and ranged from 0.38 in OarAE54 to 0.87 in MCM 140. All the 22 microsatellite loci showed high polymorphism (PIC > 0.5) for evaluating genetic diversity with the exception of BM1824 and OarAE54 (0.5 < PIC > 0.25) which is reasonably informative and included for further analysis (Table 1). Deviations from HWE were statistically significant (*P* < 0.05) for most loci except MAF33, OarJMP29, MAF214, and OarAE54 (Table 1).

**Table 1:** Total number of allele per locus (TNA) polymorphic information content (PIC), inbreeding coefficient (*F*_IS_), and observed *(H*_*O*_) and expected (*H*_E_) heterozygosity for 22 microsatellite markers analyzed in eastern Ethiopia sheep

Locus	TNA	PIC	*F*_IS_	*H*_o_	*H*_E_	HWE	Chm
BM8125	7	0.73	0.44	0.44	0.78	*	OAR 1
BM1824	3	0.44	0.36	0.34	0.52	*	OAR 1
OarCP34	9	0.73	0.30	0.55	0.78	*	OAR 3
OarAE129	9	0.55	0.48	0.32	0.63	*	OAR 5
MCM140	19	0.87	0.19	0.72	0.90	*	OAR 6
BM1329	14	0.57	0.51	0.31	0.63	*	OAR 6
ILSTS5	22	0.79	0.29	0.59	0.83	*	OAR 7
MAF33	10	0.79	− 0.03	0.85	0.83	ns	OAR 9
OarFCB193	11	0.58	0.84	0.10	0.64	*	OAR 11
HUJ616	15	0.79	0.14	0.71	0.83	*	OAR 13
MAF214	4	0.64	− 0.09	0.77	0.70	ns	OAR 16
OarHH47	19	0.82	0.18	0.70	0.86	*	OAR18
OarFCB304	18	0.83	0.41	0.51	0.87	*	OAR19
OarJMP29	11	0.76	− 0.06	0.85	0.80	ns	OAR 24
OarVH72	6	0.60	0.17	0.56	0.67	*	OAR 25
OarAE54	4	0.38	− 0.49	0.70	0.47	ns	OAR 25
OarJMP58	13	0.85	0.25	0.66	0.88	*	OAR26
SRCRSP8	23	0.83	0.12	0.76	0.86	*	NA
ILSTS087	6	0.68	0.29	0.53	0.75	*	BTA 14
SRCPSP1	13	0.61	0.18	0.53	0.65	*	CHI 13
SRCPSP9	10	0.68	0.18	0.61	0.74	*	CHI 12
ISTS011	9	0.72	0.44	0.45	0.78	*	BAT 14
Overall	255	0.69	0.24	0.57	0.75	*	

SRCRSP1, ILSTS087, ILSTS011, and SRCRSP9 are not mapped in sheep (*Ovis aries*)

*HWE* Hardy–Weinberg equilibrium, *NA* not available, *chm* chromosome number

*P < 0.05

For each population, the total and mean number of alleles, allelic richness, effective number of alleles, private alleles, observed and expected, and *F*_*IS*_ (inbreeding coefficient) are presented in Table S3. Both the highest and lowest total and mean number of alleles per population are found in the BHS populations. Sheep located in Harshin and Babile had the highest (165) and lowest (148) total number of alleles, respectively, while sheep populations located in Jijiga (7.50) and Babile (6.73) had the highest and lowest mean number of alleles, respectively. The observed heterozygosity was lower than the expected. The observed heterozygosity was ranged from 0.54 ± 0.02 in AFR (Awash), BHS (Babile), and HHL (Gorogutu) sheep to 0.62 ± 0. 02 in BHS (Harshin) sheep. Whereas, the expected heterozygosity ranged from 0.72 ± 0.04 in BHS (Shinile) to 0.76 ± 0.02 in AFR (Awash) and HHL (Deder) sheep, respectively. AFR sheep had the highest (Awash = 7.17) while BHS had the lowest (Babile = 6.73) allelic richness value (Table S3).

### Genetic differentiation and relationships among populations

The mean estimates of F-statistics obtained by jackknifing over loci and populations across ten locations are presented in Table S4. The mean total inbreeding (*F*_IT_), the inbreeding coefficient of the populations relative to all the three populations (*F*_ST_), and the within populations inbreeding coefficients (*F*_IS_) were 0.26 (0.05), 0.03 (0.01), and 0.23 (0.05), respectively (Table S4). Only four loci contributed to within-population heterozygote deficiency whereas 18 loci were contributed to the total inbreeding (*F*_IT_), indicating deficiency in heterozygotes in the total population compared to between populations. It also reflects that loci within populations are more heterogeneous than across all the populations. This is also supported by the low *F*_ST_ value (3%) and the branching pattern on the phylogenetic tree (Fig. S1) which revealed no clear clusters between populations. The analysis of molecular variance (AMOVA) result also showed that most of the genetic diversity occurred within individuals (97%) while the variability among populations and among individuals within populations contributed by 0.16 and 2.89%, respectively, to the observed genetic diversity (Table 2). There was no significant difference among the three populations, which indicated that these sheep populations (AFR, BHS, and HHL) are genetically homogeneous regardless of their phenotypic differences (Table S2).

**Table 2:** Analysis of hierarchical molecular variance (AMOVA) design and results for eastern Ethiopian sheep (average over 22 loci)

Sources of variations	DF	Sum of squares	Variance components	Percentage of variations
Among groups	2	54.59	0.01^ns^	0.16
Among populations within groups	7	171.98	0.24^*^	2.89
Among individuals within populations	288	2902.58	1.90^*^	22.5
Within individuals	298	1872.00	6.28^*^	74.46
Total	595	5001.15	8.44	

Not statistically significant (*P* > 0.05), statistically significant *P < 0.05

*ns* not statistically significant, *DF* degree of freedom

The pairwise *F*_ST_ (Table 3) values ranged from 0.011 between BHS (Babile) and HHL (Gorogutu) sheep to 0.062 between AFR (Amibara) and BHS (Shinile) sheep. The pairwise *F*_ST_ values found in the current study were low but highly significant (*P* < 0.001) except the ones between BHS (Babile) and HHL (Deder and Gorogutu) sheep. This was supported by results of the Mantel test (Fig. S3) which indicated a positive correlation between the genetic and geographical distances.

**Table 3:** Pairwise tests of differentiation between three of indigenous sheep from 22 loci. *F*_ST_ values (below the diagonal) and their significance (above the diagonal) were obtained after 100,000 permutations

Populations/locations	AFR			HHL				BHS		
	Amibara	Awash	Gewane	Deder	Gorogutu	Meta	Babile	Harshin	Jijiga	Shinile
AFR										
Amibara		***	***	***	***	***	***	***	***	***
Awash	0.024	***	***	***	***	***	***	***	***	***
Gewane	0.042	0.028	***	***	***	***	***	***	***	***
HHL										
Deder	0.039	0.012	0.028	***	***	***	ns	***	***	***
Gorogutu	0.056	0.039	0.024	0.022	***	***	ns	***	***	***
Meta	0.043	0.031	0.028	0.025	0.022	***	***	***	***	***
BHS										
Babile	0.047	0.027	0.021	0.014	0.011	0.029	***	***	***	***
Harshin	0.039	0.030	0.026	0.016	0.013	0.023	0.020	****	***	***
Jijiga	0.039	0.026	0.032	0.019	0.028	0.035	0.029	0.021	***	***
Shinile	0.062	0.044	0.043	0.036	0.035	0.017	0.039	0.035	0.051	***

Not statistically significant (*P* > 0.05), statistically significant ***P < 0.05

*ns* not statistically significant

### Population genetic structure and admixture

**Fig. 2:**
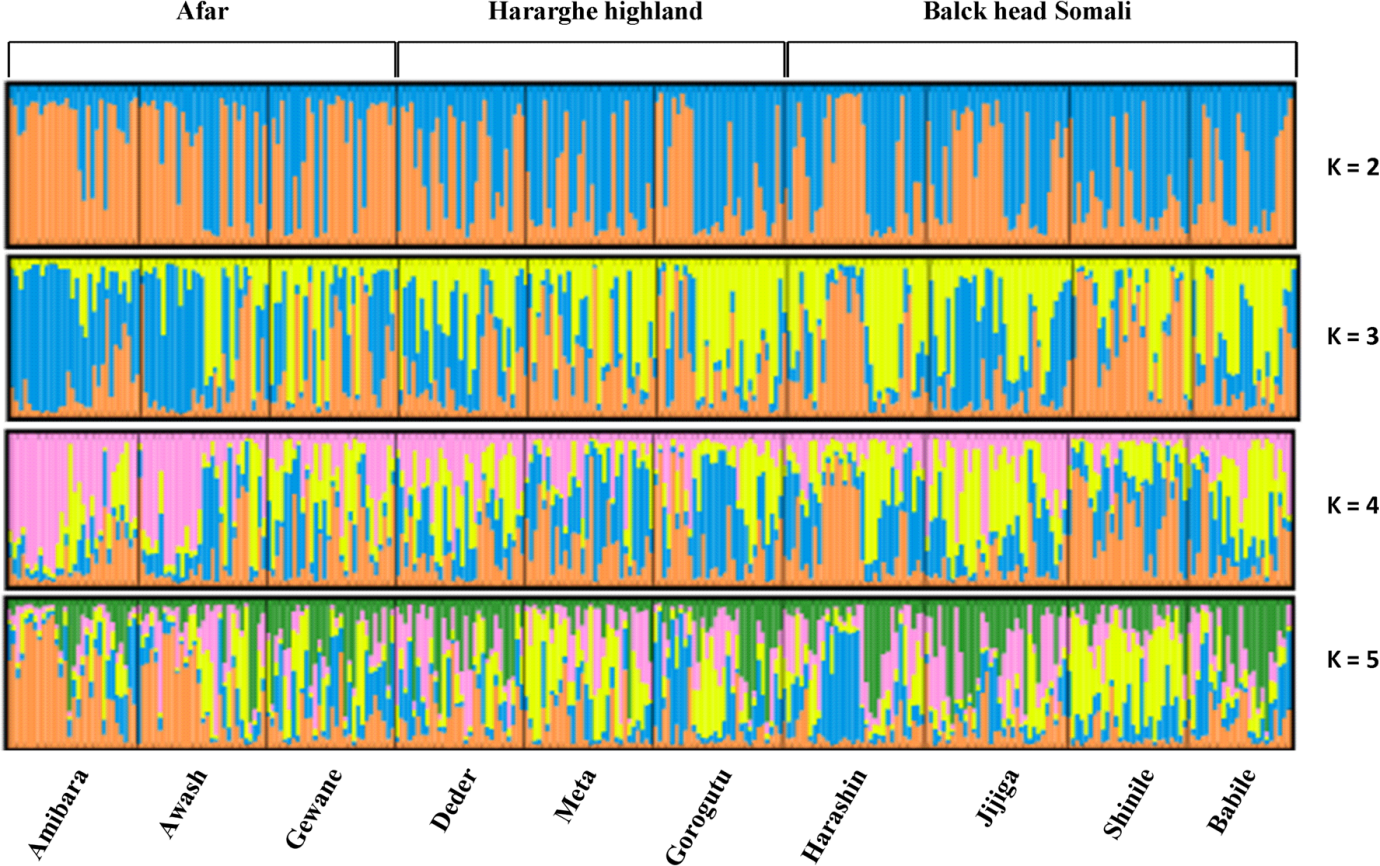
Genetic structure of three indigenous breeds of sheep and estimation of the population structure with different *K* values (assuming *K* = 2 and 5)

Graphical displays of the results from structure analysis are presented in Fig. 2. The Delta*K* approach revealed the most optimal value of *K* explaining the genetic structure and admixture in the three indigenous sheep from eastern Ethiopia was *K* = 2 (Fig. S4). At this *K* value, all the sheep populations across the ten locations show an admixed genotype of the two genetic backgrounds. However, HHL sheep shared a similar pattern from both AFR and BHS. For instance, sheep from Deder shared a pattern from AFR while the remaining sheep from Meta and Gorogutu shared a pattern from BHS sheep. At *K* = 3, *K* = 4, and *K* = 5, there was high degree of admixture between populations and it was difficult to separate one from each other except for AFR sheep located in Amibara and Awash and BHS located in Shinile which showed relatively low levels of genetic admixture compared to sheep located in other areas. These results suggest that the three indigenous populations of sheep found in eastern Ethiopia could be derived from two ancestral genetic backgrounds, the signatures of which are observed across the three populations.

### Matrilineal genetic origin and relationship among indigenous sheep populations

A total of 925 bp of the mtDNA D-loop region was sequenced in 31 samples selected from the three populations of indigenous sheep from eastern Ethiopia. We observed 137 polymorphic sites (113 parsimoniously informative and 24 singleton sites) which defined 28 haplotypes. The overall average haplotype and nucleotide diversities were 0.99 and 0.027, respectively (Table 4). The BHS sheep had the highest nucleotide diversity followed by AFR sheep. Generally, the estimates of nucleotide diversities ranged from 0.013 in HHL to 0.033 in BHS sheep (Table 4).

**Table 4:** Standard diversity indices for mtDNA analysis of eastern Ethiopian sheep breeds

Indices	AFR	HHL	BHS	Overall
Number of sample	14	8	9	31
Number of haplotypes	14	6	8	28
Number of polymorphic sites	72	19	63	99
Haplotype diversity	0.99	1.00	1.00	0.99
Nucleotide diversity	0.028	0.014	0.033	0.027
Average number of nucleotide differences	17.41	8.60	21.00	16.94

**Fig. 3:**
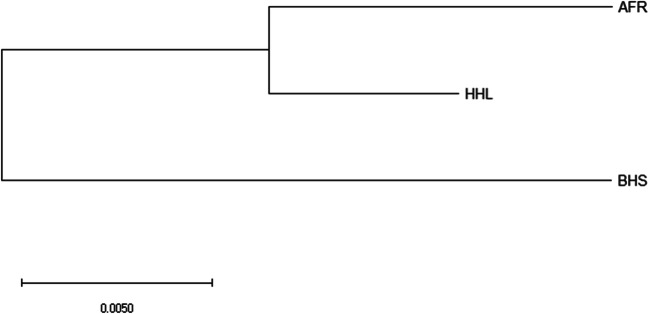
A neighbor-joining (NJ) tree of eastern Ethiopian indigenous sheep mtDNA D-loop sequences

**Fig. 4:**
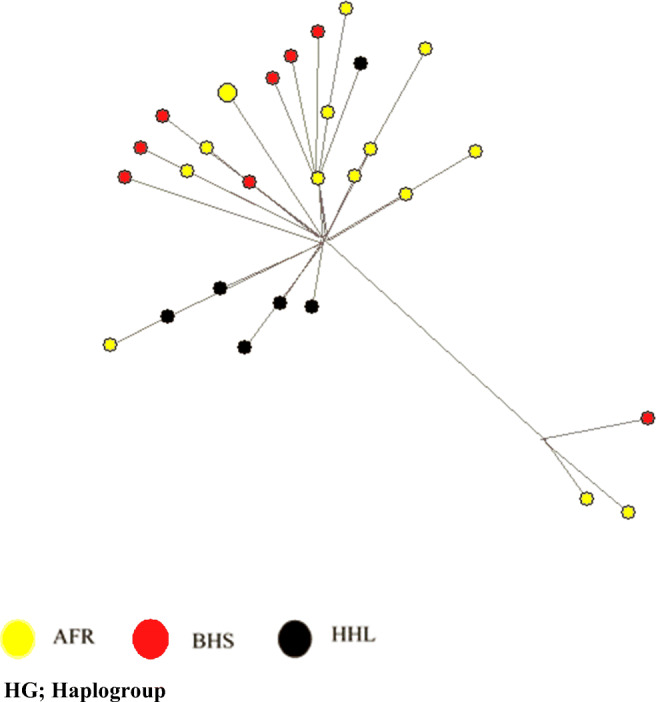
Median-joining network of the sheep mtDNA D-loop sequences

**Fig. 5:**
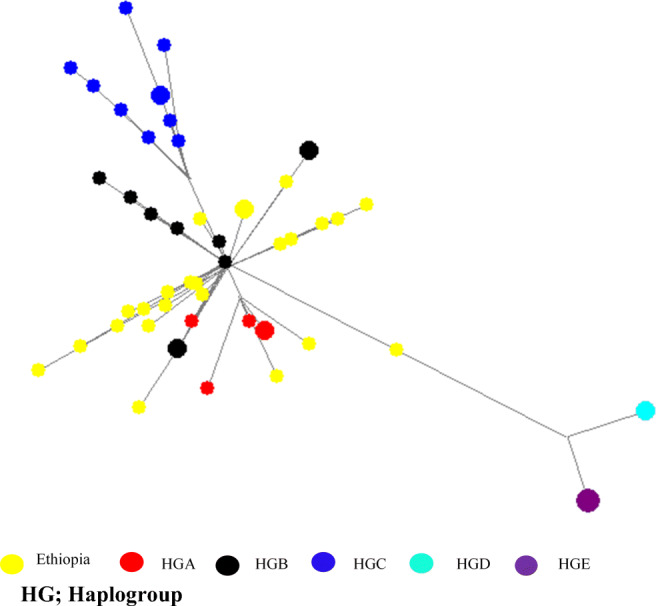
Median-joining network of mtDNA D-loop sequences haplogroups belonging to Ethiopian sheep and five haplotypes identified in domestic sheep (*Ovis aries*)

AMOVA revealed negligible inter-population genetic structuring in indigenous sheep populations (Table 5). Most of the mtDNA variations (97.72%) were accounted for intra-populations genetic structuring while the remaining (2.29%) was accounted by among populations within sheep populations. A neighbor-joining (NJ) of eastern Ethiopia sheep mtDNA sequences is shown in Fig. 3. The tree shows that all the populations originated from a common source. However, BHS diverged first and followed by AFR and HHL sheep (Fig. 3). This result is supported by median-joining network of mtDNA haplotypes in which there were two major groups (Fig. 4). All mtDNA sequences were compared with sequences of five haplotypes representing the five haplogroups (A, B, C, D, and E) that have been observed in world domestic sheep (Fig. 5). Out of the 31 mtDNA D-loop sequences generated in this study, only five (16.12%) were of haplogroup A and the rest 26 (83.88%) were of haplogroup B (Table 6). No other representative haplotypes were present in indigenous sheep from eastern Ethiopia (Fig. 5).

**Table 5:** Analysis of hierarchical molecular variance (AMOVA) design and result for eastern Ethiopian sheep (average over 28 haplotypes)

Sources of variations	DF	Sum of squares	Variance components	Percentage of variations
Among populations	2	20.34	0.20^ns^	97.72
Within populations	26	218.80	8.42^*^	2.29
Total	28	239.14	8.61	

Not statistically significant (*P* > 0.05), statistically significant *P < 0.05

*ns* not statistically significant, *DF* degree of freedom

**Table 6:** Distribution of haplotypes of haplogroups A and B among the three populations of indigenous sheep from eastern Ethiopia

Breeds	n	Type A	Type B	Type C	Type D	Type E
AFR	14	3 (9.69%)	11 (35.48%)	–	–	–
BHS	9	1 (3.22%)	8 (25.82%)	–	–	–
HHL	8	1 (3.22%)	7 (22.58%)	–	–	–
Total	31	5 (16.12%)	26 (83.88%)	–	–	–

## Discussion

### Genetic diversity

Knowledge on genetic diversity, population structure, and genetic relationships among populations are essential to design cost-effective and sustainable genetic improvement and conservation strategy. In this study, the autosomal genetic diversity and structure of three indigenous fat-rumped sheep populations from ten locations across eastern Ethiopia was assessed using 22 microsatellite markers. Additionally, the matrilineal genetic origins and relationship of the three populations were also assessed by sequencing of the mtDNA D-loop region from 31 samples.

The level of variation depicted by the number of alleles at each locus serves as a measure of the impact of each of the studied breeds on differentiation within livestock populations (Dashab et al. 2011). In the current study, the number of alleles per locus was ranged from 3 to 23, which can be considered high when compared with other studies that assessed these markers in ovine breeds (Crispim et al. 2014; Jawasreh et al. 2018; Agaviezor et al. 2012). Most of the microsatellite markers used in this study were highly polymorphic and relevant for genetic diversity study. However, most of them showed significant deviation from HWE except for MAF33, OarJMP29, MAF214, and OarAE54 markers. This may indicate migration or high mutation rate in microsatellites. It also could be due to artificial selection in the studied breeds (Dashab et al. 2011). Other factors that may affect HWE values include inbreeding, selection, mutation, and migration. Deviation from HWE at different marker loci has been reported previously in various breeds of sheep (Dashab et al. 2011; Abdelkader et al. 2018; Jawasreh et al. 2018).

Genetic variability is also measured as the amount of actual or potential heterozygosity. Genetic variability within the ten sheep populations is high, as evidenced by the high mean expected heterozygosity (*H*_E_). The range in values for *H*_E_ and *H*_O_ observed in this study compare favorably with previously published data for other African sheep populations (Gizaw et al. 2007; Muigai et al. 2009; Gornas et al. 2011; Agaviezor et al. 2012; Ben Sassi-Zaidy et al. 2014; and Gaouar et al. 2016). The observed heterozygosity was lower than the expected heterozygosity in the three populations across all loci which indicated increasing homozygosity in most sheep populations. The difference between the observed and expected heterozygosity can be attributed to the non-random mating among the individuals of the population. This was also reflected in the positive *F*_IS_ value (0.24) which ranged from − 0.49 to 0.87. The difference between the observed and expected heterozygosity can be attributed to the non-random mating among the individuals of the population. Similar to our assumptions, the high *F*_IS_ value (0.302 ± 0.057) in the Tibetan sheep was considered as the major cause of the Hardy–Weinberg disequilibrium in that population (Sharma et al. 2016). The values of allelic richness observed in eastern Ethiopia sheep are comparable with those reported previously for Ethiopian sheep (6.79) by Gizaw et al. (2007); they are lower than those reported in Nigerian sheep (8.63) (Agaviezor et al. 2012). Measures of genetic diversity based on allelic richness are considered important in conservation genetics because marker-assisted methods for maximizing the number of alleles conserved have been shown to be effective (Bataillon et al. 1996). Allelic richness may be a useful indicator of a decrease in population size or of past bottlenecks (Agaviezor et al. 2012).

The overall *F*_ST_ value and the AMOVA result revealed little, but a highly significant level of genetic differentiation among the study sheep populations. Approximately 3% of the total genetic variation could be attributed to differences among populations, the remaining 97% being accounted for by differentiation among individuals within sheep populations. This might be due to migration of individuals from one population to the others as evidenced by the phylogenetic tree in which there was no clear cluster formed between populations. Exchanging breeding ram and animal movement from one population to the other is common practice in eastern Ethiopia (Nigussie et al. 2013). On the other hand, Dadi et al. (2008) and Hassen et al. (2012) indicated that traditional uncontrolled mating practices and movement of animals through various market routes and agricultural extension systems in Ethiopia could be one of the reasons for low genetic differentiation between populations. Gizaw et al. (2007) and Gaouar et al. (2016) also confirmed that lack of differentiation in those phenotypically different populations could be due to gene flow between the areas having close geographical distance and similar ecology and large population size. The pairwise *F*_ST_ values indicated a close genetic relationship between BHS sheep located in Babile and HHL sheep located in Gorogutu and Deder. This observation might be as a result of gene flow due to the close geographical proximity between sheep across the three locations. On the other hand, AFR sheep located in Amibara and BHS sheep located in Shinile were the most genetically differentiated populations. In this data, we observed a positive relationship between genetic and geographic distances. Sheep populations that are found in proximity are closely genetically related compared to the ones that are separated by larger genetic distances.

### Population structure and admixture

Results from the STRUCTURE analysis revealed that varying the number of presumed ancestral populations (*K*) produces clusters that are consistent with matrilineal genetic origin of eastern Ethiopia sheep. The first level of clustering (*K* = 2), which is revealed by DeltaK to be the most optimal, reflects that three sheep populations across ten locations derived from two ancestral populations (gene pool). The first genetic background predominated in sheep populations of AFR while the second background occurred at higher frequency in the BHS. All the BHS and AFR sheep across all locations clustered within their respective population groups, whereas the HHL sheep were grouped in both clusters. For example, sheep located in Deder shared a similar pattern with AFR sheep but Meta and Gorogutu shared the same pattern with BHS Sheep. This, therefore, HHL sheep may not be a pure/independent population, rather they are genetically an admixture with both AFR and BHS sheep. This result is in an agreement with the result of Nigussie et al. (2016) who indicated that HHL sheep shared common phenotypic characteristics with both AFR and BHS sheep. Besides, owners of HHL sheep confirmed that their sheep do not have a uniform coat color and tail type (Nigussie et al. 2015). Further evaluation of the clusters, at *K* = 3, *K* = 4, and *K* = 5, revealed the presence of sub-clusters and high degree of genetic admixture which are indicative of substantial gene flow between sheep populations. However, AFR sheep found in Amibara and BHS found in Shinile are the only sheep populations that showed a relatively low level of admixture which is supported by relatively high *F*_ST_ value between them. Nigussie et al. (2016) indicated that the genetic variation of eastern Ethiopia sheep might be the result of agro-ecological similarity, adaptation to environments where they are managed in addition to the breeding practices of sheep owners. However, controlled breeding should be practiced by avoiding individual animal movement and indiscriminate breeding to maintain the purity of each population.

### Matrilineal genetic origin and relationship of eastern Ethiopia populations

As revealed in several genetic diversity studies (e.g., Meadows et al. 2005; Agaviezor et al. 2012; Lancioni et al. 2013), the mtDNA D-loop region provides sufficient evidence to assess population genetic diversity, evolutionary relationships, and matrilineal genetic origin of species. The present study provides the first insights on the maternal evolutionary relationships among AFR, BHS, and HHL sheep and their possible maternal origins and affiliations to other global sheep populations.

The observed haplotype and nucleotide diversity of the three eastern Ethiopia sheep was higher than that reported by (Agaviezor et al. 2012) for Nigerian sheep and (Oner et al. 2013) for Turkish sheep. The highest nucleotide diversity observed in AFR and BHS might be due to their distribution across different agro-ecology and production environments. They are the most numerous sheep populations in eastern Ethiopia compared to HHL sheep. The NJ tree separated the three sheep populations into two major groups which are evidenced by autosomal DNA analysis. However, clustering of HHL sheep is not the same with mtDNA (HHL with AFR) and autosomal DNA analysis (HHL with BHS). Hence, the most probable genetic origin of HHL sheep is AFR sheep though there was genetic intermixing with BHS sheep. The phylogenetic tree also showed that there is close maternal genetic relationship between AFR and HHL sheep compared to BHS sheep.

The mtDNA analysis revealed the presence of two haplogroups (A and B) in eastern Ethiopia sheep though Haplogroup B occurs at higher frequency than haplogroup (A). This result in a way corresponds to that of the STRUCTURE analysis which revealed the most optimal *K* to be two indicating the presence of two ancestral genetic backgrounds. Similar to the two autosomal genetic backgrounds, the two mtDNA haplogroups also showed no phylogeographic structure suggesting extensive intermixing. The current result of both microsatellite and the mtDNA appear to support the fact that indigenous sheep from eastern Ethiopia derive from two ancestral genetic backgrounds. The two genetic backgrounds might have evolved independently before being introduced into the country. However, it is unclear whether these two backgrounds were introduced simultaneously into Ethiopia or independent of each other and at different time periods. The current result was in agreement with the result of Gornas et al. (2011) who reported the presence of haplogroups B and A in Sudanese sheep. Haplogroup B also predominates in sheep from East, West, and South Africa (Resende et al. 2016; Agaviezor et al. 2012; Ann Horsburgh and Rhines 2010, respectively). Haplogroup A, however, is common in Asian and Arabian sheep breeds. Muigai and Hanotte (2013) pointed that African sheep share a common maternal ancestry with European and Asian sheep, and that they likely originated from the same center(s) of domestication. The first sheep in east Africa were introduced directly from the Arabian Peninsula (Muigai and Hanotte 2013). Undated rock paintings from Serkama Cave (Harar Province) in the eastern Ethiopia highlands (Clark and Williams 1978) suggest that fat-tailed sheep were present in Ethiopia and east Africa, for which there is undisputed archeological evidence dated to around the mid-first millennium AD (Gifford-Gonzalez and Hanotte 2011). However, there is limited information on the matrilineal genetic origin of sheep in Africa in general and in east Africa in particular.

## Conclusion

The current result indicated that the within genetic diversity is high than between populations, which will be an opportunity to design a cost-effective and sustainable genetic improvement and conservation program for eastern Ethiopia sheep. Both autosomal microsatellite markers and mtDNA D-loop sequences diversity analysis revealed that eastern Ethiopia sheep breeds most likely derive from two ancestral genetic backgrounds. HHL sheep had high genetic admixture with both AFR and BHS though mtDNA D-loop sequences analysis indicated that the sheep clustered with AFR sheep population. These findings need to be confirmed with the analysis of more populations from adjoining area to address the breeding tract of HHL sheep. The result for the matrilineal genetic origin of indigenous sheep is preliminary; therefore, more samples from wider geographical location and east African countries are required to substantiate the evolutionary history of Ethiopian sheep.

## Electronic supplementary material

ESM 1 (DOCX 872 kb)
