# Supplementary material for: Genetic diversity and matrilineal genetic origin of fat-rumped sheep in Ethiopia
Source: Trop Anim Health Prod. 2019 Feb 24;51(6):1393–404. doi: 10.1007/s11250-019-01827-z (PMC7826308; doi:10.1007/s11250-019-01827-z)
Supplement: Supplementary file 1 — (DOCX 872 kb) [file 11250_2019_1827_MOESM1_ESM.docx]

Supplement

**Table S1** Description of sampled sheep breeds/populations

| Breeds | Feature and use | location | Agro ecology | Production system |
| --- | --- | --- | --- | --- |
| Afar | Coat color varies from white to light red; straight head profile; fat rumped/ fat tailed, and the tail was curved upward having a wider tail both at the base and at the tip; used for milk production, income generation and mutton production. | Awash | Arid | Pastoral |
|  |  | Amibara | Arid | Agro-pastoral |
|  |  | Gewane | Arid | Pastoral |
| Black Head Somali | Black head with white body color; body covered with short smooth and rough hair; Fat rumped tail and straight head profile; used for milk production, income generation and mutton production. | Jijiga | Semi-arid | Agro-pastoral |
|  |  | Harshin | Semi-Arid | Pastoral |
|  |  | Shinile | Semi-arid | Pastoral |
|  |  | Babile | Mid land | Agro-pastoral |
| Hararghe Highland | Coat color light brown with white patch in females and white in males; the body was covered with short smooth /rough hair; fat tailed; females had straight head profile while majority of males had convex head profile; income generation and mutton production. | Deder | Highland | Crop-livestock mixed |
|  |  | Gorogutu | Highland | Crop-livestock mixed |
|  |  | Meta | Highland | Crop-livestock mixed |

**Table S2** Microsatellite primer sequences, chromosome number (Chm), annealing temperature (AT), that were used to study eastern Ethiopian sheep

| **S.N** | **Name** | **sequence (5' to 3')** | **Chn** | **AT** | **dye** |
| --- | --- | --- | --- | --- | --- |
| 1 | OarHH47 | TTTATTGACAAACTCTCTTCCTAACTCCACC | OAR 18 | 55-60 | VIC |
|  |  | GTAGTTATTTAAAAAAATATCATACCTCTTAAGG |  |  |  |
| 2 | OarVH72 | GGCCTCTCAAGGGGCAAGAGCAGG | OAR 25 | 55-60 | NED |
|  |  | CTCTAGAGGATCTGGAATGCAAAGCTC |  |  |  |
| 3 | OarAE129 | AATCCAGTGTGTGAAAGACTAATCCAG | OAR 5 | 55-60 | PET |
|  |  | GTAGATCAAGATATAGAATATTTTTCAACACC |  |  |  |
| 4 | BM1329 | TTGTTTAGGCAAGTCCAAAGTC | OAR 6 | 55-60 | 6-FAM |
|  |  | AACACCGCAGCTTCATCC |  |  |  |
| 5 | OarFCB193 | TTCATCTCAGACTGGGATTCAGAAAGGC | OAR 11 | 55-60 | PET |
|  |  | GCTTGGAAATAACCCTCCTGCATCCC |  |  |  |
| 6 | OarJMP58 | GAAGTCATTGAGGGGTCGCTAACC | OAR 26 | 50-60 | 6-FAM |
|  |  | CTTCATGTTCACAGGACTTTCTCTG |  |  |  |
| 7 | MAF214 | GGGTGATCTTAGGGAGGTTTTGGAGG | OAR16 | 50-60 | VIC |
|  |  | AATGCAGGAGATCTGAGGCAGGGACG |  |  |  |
| 8 | BM1824 | GAGCAAGGTGTTTTTCCAATC | OAR1 | 60-65 | NED |
|  |  | CATTCTCCAACTGCTTCCTTG |  |  |  |
| 9 | OarJMP29 | GTATACACGTGGACACCGCTTTGTAC | OAR 24 | 55-60 | VIC |
|  |  | GAAGTGGCAAGATTCAGAGGGGAAG |  |  |  |
| 10 | OarFCB304 | CCCTAGGAGCTTTCAATAAAGAATCGG | OAR 19 | 55-60 | NED |
|  |  | CGCTGCTGTCAACTGGGTCAGGG |  |  |  |
| 11 | OarCP34 | GCTGAACAATGTGATATGTTCAGG | OAR 3 | 60-65 | 6-FAM |
|  |  | GGGACAATACTGTCTTAGATGCTGC |  |  |  |
| 12 | SRCRSP1 | TGCAAGAAGTTTTTCCAGAGC | CHI13 | 55-65 | PET |
|  |  | ACCCTGGTTTCACAAAAGG |  |  |  |
| 13 | HUJ616 | TTCAAACTACACATTGACAGGG | OAR13 | 55-60 | NED |
|  |  | GGACCTTTGGCAATGGAAGG |  |  |  |
| 14 | MAF33 | GATCTTTGTTTCAATCTATTCCAATTTC | OAR9 | 50-65 | 6-FAM |
|  |  | GATCATCTGAGTGTGAGTATATACAG |  |  |  |
| 15 | MCM140 | GTTCGTACTTCTGGGTACTGGTCTC | 0AR6 | 60-65 | PET |
|  |  | GTCCATGGATTTGCAGAGTCAG |  |  |  |
| 16 | BM8125 | CTCTATCTGTGGAAAAGGTGGG | OAR17 | 60-65 | VIC |
|  |  | GGGGGTTAGACTTCAACATACG |  |  |  |
| 17 | SRCRSP8 | TGCGGTCTGGTTCTGATTTCAC |  | 55-65 | PET |
|  |  | GTTTCTTCCTGCATGAGAAAGTCGATGCTTAG |  |  |  |
| 18 | ILSTS087 | AGCAGACATGATGACTCAGC | BTA6 | 55-65 | NED |
|  |  | CTGCCTCTTTTCTTGAGAG |  |  |  |
| 19 | OarAE54 | TACTAAAGAAACATGAAGCTCCCA | OAR25 | 55-60 | VIC |
|  |  | GGAAACATTTATTCTTATTCCTCAGTG |  |  |  |
| 20 | ILSTS011 | GCTTGCTACATGGAAAGTGC | BTA14 | 55-65 | PET |
|  |  | CTAAAATGCAGAGCCCTACC |  |  |  |
| 21 | ILSTS5 | GGAAGCAATGAAATCTATAGCC | OAR7 | 55-60 | VIC |
|  |  | TGTTCTGTGAGTTTGTAAGC |  |  |  |
| 22 | SRCRSP9 | AGAGGATCTGGAAATGGAATC | CHI12 | 50-55 | 6-FAM |
|  |  | GCACTCTTTTCAGCCCTAATG |  |  |  |

SRCRSP1, ILSTS087, ILSTS011 and SRCRSP9 are not mapped in *Ovis aries*; Chn, chromosome number, AT; annealing temperature

**Table S3** Indicators of genetic diversity in indigenous sheep breeds of eastern Ethiopia analyzed using 22 microsatellite markers

| Populations/Locations | N | TNA | MNA(±SD) | AR | | ENA | | H_E_ (±SD) | | H_o_(±SD) | | *F_IS_* | |  |
| --- | --- | --- | --- | --- | --- | --- | --- | --- | --- | --- | --- | --- | --- | --- |
| AFR |  |  |  |  | |  | |  | |  | |  | |  |
| Amibara | 30 | 159 | 7.23(3.31) | 6.96 | | 4.04 | | 0.74(0.02) | | 0.60(0.02) | | 0.20^*^ | |  |
| Awash | 30 | 163 | 7.41(3.62) | 7.17 | | 4.54 | | 0.76(0.02) | | 0.54(0.02) | | 0.29^*^ | |  |
| Gewane | 30 | 162 | 7.36(3.14) | 7.09 | | 4.39 | | 0.74(0.03) | | 0.56(0.02) | | 0.25^*^ | |  |
| Overall | 30 | 115 | 7.33(3.14) | 7.07 | | 4.32 | | 0.75(0.02) | | 0.57(0.02) | | 0.25^*^ | |  |
| HHL |  |  |  |  | |  | |  | |  | |  | |  |
| Deder | 30 | 159 | 7.23(3.12) | 7.00 | | 4.60 | | 0.76(0.03) | | 0.60(0.02) | | 0.21^*^ | |  |
| Gorogutu | 30 | 158 | 7.18(3.53) | 6.92 | | 4.44 | | 0.73(0.03) | | 0.54(0.02) | | 0.26^*^ | |  |
| Meta | 30 | 160 | 7.27(3.48) | 7.04 | | 4.65 | | 0.74(0.03) | | 0.58(0.02) | | 0.23^*^ | |  |
| Overall | 30 | 118 | 7.29(3.36) | 6.99 | | 4.56 | | 0.75(0.03) | | 0.57(0.02) | | 0.23^*^ | |  |
| BHS |  |  |  |  | |  | |  | |  | |  | |  |
| Babile | 24 | 148 | 6.73(3.45) | 6.73 | | 4.51 | | 0.73(0.03) | | 0.54(0.02) | | 0.27^*^ | |  |
| Harshin | 33 | 165 | 7.14(2.87) | 6.84 | | 4.77 | | 0.75(0.03) | | 0.61(0.02) | | 0.19^*^ | |  |
| Jijiga | 33 | 157 | 7.50(3.57) | 7.05 | | 4.54 | | 0.75(0.03) | | 0.58(0.02) | | 0.24^*^ | |  |
| Shinile | 30 | 155 | 7.05(3.14) | 6.86 | | 4.37 | | 0.72(0.04) | | 0.56(0.02) | | 0.22^*^ | |  |
| Overall | 30 | 128 | 7.10(3.26) | 6.87 | | 4.55 | | 0.75(0.02) | | 0.57(0.02) | | 0.23^*^ | |  |
| Across eastern Ethiopia | 300 | 255 | 7.21(3.32) | | 6.97 | 4.49 | 0.75(0.04) | | 0.57(0.19) | | 0.23 | |  | |
| ^N = Sample size; TNA = Total number of alleles; MNA = Mean number of alleles; AR = Allelic richness; ENA = Effective number of alleles; H^_E_ ^= Expected heterozygosity; H^_o_ ^= Observed heterozygosity; SD = Standard deviation;^ *^F^_IS_* ^= Coefficient of inbreeding (significant values are as indicated *P < 0.05).^ | | | | | | | | | | | | | | |

Table S4 Result of F-statistics for each locus in the overall eastern Ethiopia sheep breeds obtained by jackknifing

| Locus | *F_IT_ (±SE)* | *F_ST_(±SE)* | *F_IS_(±SE)* |
| --- | --- | --- | --- |
| OarFCB304 | 0.43(0.04) ^*^ | 0.03(0.02)^*^ | 0.42(0.04)^*^ |
| OarFCB193 | 0.86(0.05) ^*^ | 0.08(0.04)^*^ | 0.85(0.06)^*^ |
| OarCP34 | 0.31(0.04) ^*^ | 0.01(0.01)^ns^ | 0.30(0.04)^*^ |
| MCM140 | 0.20(0.03) ^*^ | 0.01(0.01)^ns^ | 0.19(0.02)^*^ |
| OarHH47 | 0.21(0.05) ^*^ | 0.03(0.01) ^*^ | 0.19(0.05)^*^ |
| OarJMP58 | 0.25(0.04) ^*^ | 0.01(0.00)^ns^ | 0.25(0.04)^*^ |
| BM8125 | 0.45(0.05) ^*^ | 0.00(0.01)^ns^ | 0.45(0.05)^*^ |
| MAF33 | -0.03(0.05)^ns^ | 0.01(0.01)^ns^ | -0.04(0.04)^ns^ |
| OarJMP29 | -0.05(0.03)^ns^ | 0.01(0.00)^ns^ | -0.06(0.03)^ns^ |
| ILSTS087 | 0.27(0.06) ^*^ | 0.00(0.01)^ns^ | 0.28(0.06) ^*^ |
| SRCRSP8 | 0.14(0.05) ^*^ | 0.02(0.01) ^*^ | 0.12(0.04)^ns^ |
| MAF214 | -0.08(0.04)^ns^ | 0.02(0.01) ^*^ | -0.10(0.03)^ns^ |
| SRCPSP1 | 0.22(0.05) ^*^ | 0.06(0.02) ^*^ | 0.189(0.05) ^*^ |
| SRCPSP9 | 0.19(0.06) ^*^ | 0.02(0.01) ^*^ | 0.17(0.06) ^*^ |
| OarVH72 | 0.21(0.10) ^*^ | 0.06(0.02) ^*^ | 0.17(0.10) ^*^ |
| BM1329 | 0.53(0.07) ^*^ | 0.05(0.02) ^*^ | 0.51(0.07)^ns^ |
| ISTS011 | 0.44(0.05) ^*^ | 0.02(0.01) ^*^ | 0.43(0.05) ^*^ |
| ILSTS5 | 0.32(0.05) ^*^ | 0.05(0.02) ^*^ | 0.29(0.04) ^*^ |
| HUJ616 | 0.16(0.04) ^*^ | 0.02(0.01) ^*^ | 0.14(0.05) ^*^ |
| OarAE129 | 0.54(0.05) ^*^ | 0.08(0.02) ^*^ | 0.50(0.06) ^*^ |
| BM1824 | 0.40(0.06) ^*^ | 0.07(0.04) ^*^ | 0.36(0.07) ^*^ |
| OarAE54 | -0.39(0.08)^ns^ | 0.08(0.03) ^*^ | -0.52(0.05) ^ns^ |
| Overall | 0.26(0.05) | 0.03(0.01) | 0.23(0.05) |

^ns; not statistically significant (P > 0.05) , statistically significant, * P < 0.05^


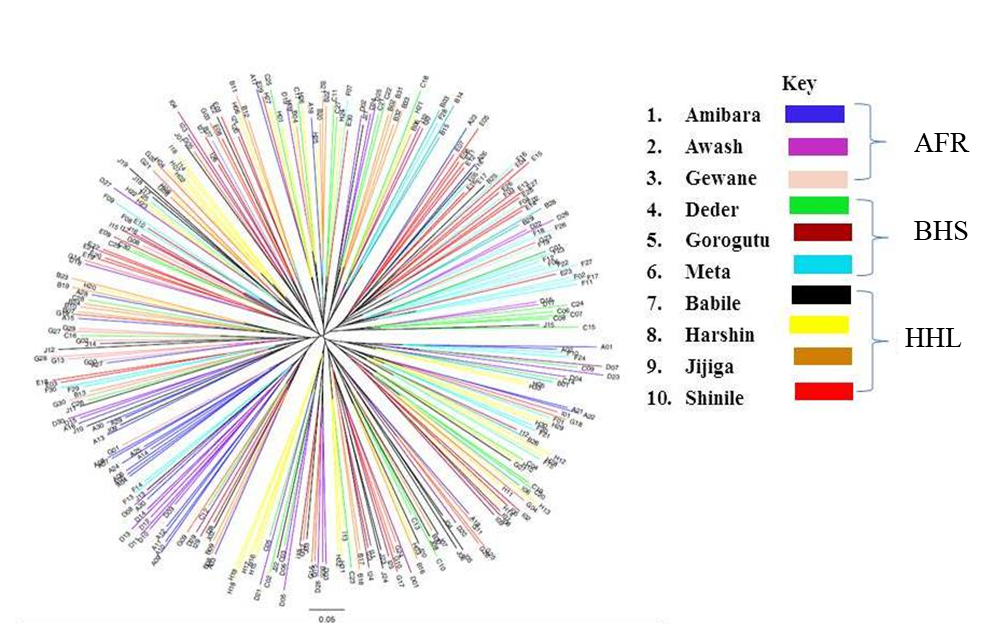


**Fig. S1** Phylogenic tree showing genetic relationship among three breeds of sheep across to locations of eastern Ethiopia


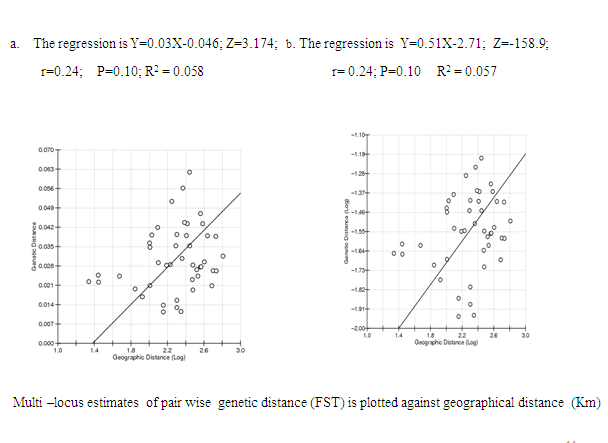


**Fig.S2** Multi-locus estimates of pairwise genetic distance (*F_ST_*) is plotted against geopgraphical distance(Km)


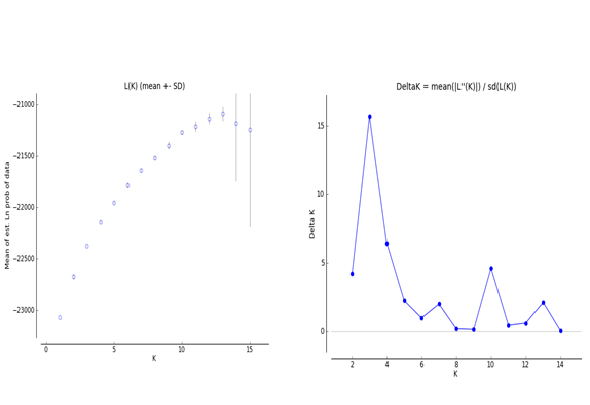


**Fig S3** Optimum K value from STRUCTURE analysis following Evanno et al. (2005)
